# Supplementary material for: Vitamin D Modified DSS-Induced Colitis in Mice via STING Signaling Pathway
Source: Biology (Basel). 2025 Jun 18;14(6):715. doi: 10.3390/biology14060715 (PMC12190092; doi:10.3390/biology14060715)
Supplement: Supplementary file 1 [file biology-14-00715-s001.zip › Supplementary Materials S1: 16s-report of gut microbiota/Groups/diff_analysis/UnivarTestGenus/figure/boxplot/top10log.pdf]

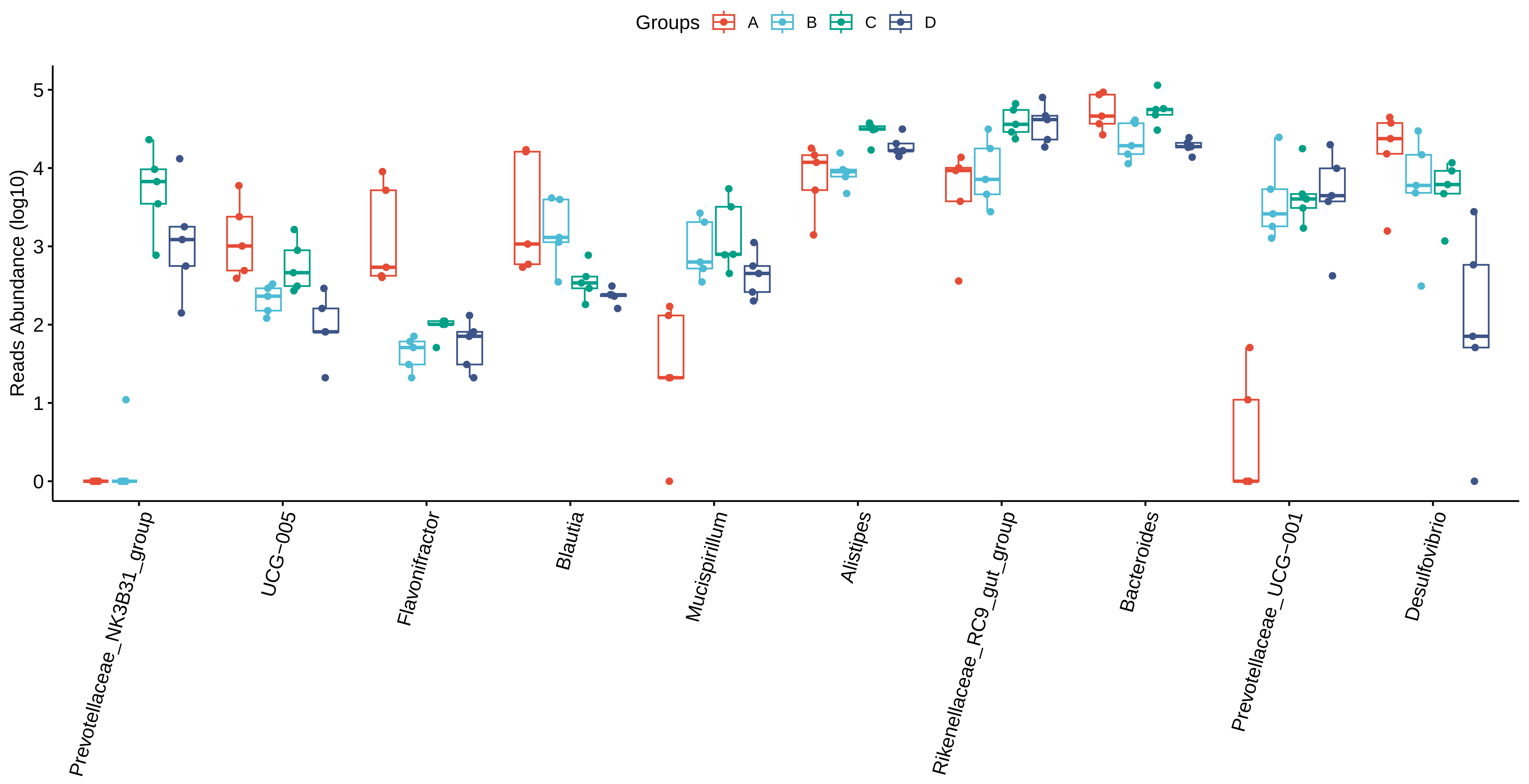

Groups A B C D
Reads Abundance (log10)
0
1
2
3
4
5
Prevotellaceae_NK3B31_group
UCG−005
Flavonifractor
Blautia
Mucispirillum
Alistipes
Rikenellaceae_RC9_gut_group
Bacteroides
Prevotellaceae_UCG−001
Desulfovibrio
